# Effects of conditioning activity mode, rest interval and effort to pause ratio on post-activation performance enhancement in taekwondo: a randomized study

**DOI:** 10.3389/fphys.2023.1179309

**Published:** 2023-07-12

**Authors:** Ibrahim Ouergui, Slaheddine Delleli, Hamdi Messaoudi, Craig Alan Bridge, Hamdi Chtourou, Emerson Franchini, Luca Paolo Ardigò

**Affiliations:** ^1^ High Institute of Sport and Physical Education of Kef, University of Jendouba, Jendouba, Tunisia; ^2^ Research Unit: Sport Sciences, Health and Movement, UR22JS01, University of Jendouba, El Kef, Tunisia; ^3^ High Institute of Sport and Physical Education of Sfax, University of Sfax, Sfax, Tunisia; ^4^ Research Unit Physical Activity, Sport and Health, National Observatory of Sport, Tunis, Tunisia; ^5^ Sports Performance Research Group, Wilson Centre, Edge Hill University, Ormskirk, United Kingdom; ^6^ Martial Arts and Combat Sports Research Group, School of Physical Education and Sport, University of São Paulo, São Paulo, Brazil; ^7^ Department of Teacher Education, NLA University College, Oslo, Norway

**Keywords:** potentiation, plyometrics, repeated techniques, combat sports, expert athletes potentiation, expert athletes

## Abstract

**Introduction:** The present study assessed the effects of conditioning activities, using different effort-to-pause ratios and rest intervals, on taekwondo physical performance.

**Methods:** Twenty-one athletes (13 males and 8 females) (Mean ± SD; age = 20.4 ± 1.4 years) performed a control (CC) and twelve experimental conditions. Each condition contained a standard warm-up (i.e., CC: running at 9 km/h for 10 min) and conditioning activities comprising plyometrics P) or repeated high-intensity techniques (RT) using 1:6, 1:9 and self-selected rest (SSR) ratios, and two rest intervals (3 and 7 min). Athletes then performed a battery of fitness tests: countermovement jump (CMJ), taekwondo specific agility (TSAT), 10s and multiple frequency speed kick test (FSKT-10s and FSKT-mult, respectively).

**Results:** All of the preloads provided higher performance outputs compared to the control trial (all *p* < 0.05). For CMJ, 1:6 ratio with 3 min induced lower values with RT compared to P (*p* = 0.037) and 1:9 ratio using 3 min induced higher values with RT compared to P (*p* = 0.027). Additionally, 1:6 ratio using 7 min induced higher values with RT compared to P (*p* = 0.016). For FSKT-10, 3 min using 1:6 induced higher values with P compared to RT, while RT induced higher values with 7 min using 1:6 ratio compared to P (both *p* < 0.001). Moreover, 3 min using 1:9 ratio induced higher values with P compared to RT (*p* = 0.034), while RT induced higher values with 1:9 ratio using 7 min compared to P (*p* < 0.001). Finally, 3 min using SSR ratio induced higher values with RT compared to P (*p* = 0.034).

**Conclusion:** Plyometrics and RT activities improved performance with plyometrics requiring shorter rest interval to induce potentiation effects compared to RT, which required longer interval.

## 1 Introduction

The ability to generate power in the lower limbs is crucial for success in numerous athletic events (Santos et al., 2021). Taekwondo is a high-intensity intermittent striking combat sport, which requires speed, agility and the execution of powerful kicks to succeed in competition (Bridge et al., 2014). The warm-up is an integral part of athletes’ training and competition preparations, and it may facilitate performance through adjustments in several temperature and non-temperature related mechanism (Blazevich and Babault, 2019; Dobbs et al., 2019). In this regard, different strategies have been incorporated into the warm-up aiming to acutely augment athletes’ peak power production (Dobbs et al., 2019). Among numerous strategies, voluntary muscular contractions at maximal or near-maximal intensities have been used during the warm-up as a conditioning activity (CA) (Dobbs et al., 2019). The performance enhancement recorded after a high-intensity CA is known as post-activation performance enhancement (PAPE) (Blazevich and Babault, 2019; Boullosa et al., 2020). PAPE refers to the phenomen in which “high-intensity voluntary conditioning contraction(s) enhance voluntary muscular performance in a subsequent voluntary contraction rather than electrically evoked (twitch), force production” (Blazevich and Babault, 2019, p. 04). This phenomenon appears when physiological mechanisms of potentiation (e.g., increase in spinal-level excitability, muscle twitch response, and muscle-tendon stiffness) dominates fatigue produced at the same time (Seitz and Haff, 2016). In addition, the mechanisms of PAPE generation include an increase in the rate of force production, muscular activation, muscle temperature, blood flow, intracellular water accumulation, and reduced hypotonicity (Blazevich and Babault, 2019).

Various forms of high-intensity CA can enhance power output (Dobbs et al., 2019). This includes resistance, isometric, plyometric, resisted sprint, and combined exercises (Ng et al., 2020). Whilst the use of different types of CA in training does not present many issues, applying it in competition settings is more challenging due to the equipment limitations and timing restrictions (Kilduff et al., 2013). For this reason, it seems that ballistic exercises based on easy-to-use equipment (i.e., the lack of onerous equipment requirements), such as the application of plyometric (Kilduff et al., 2013; Maloney et al., 2014), or sport-specific technical activities, may be more suitable in competition (Aandahl et al., 2018). In this context, performing upper and lower body plyometric exercises before the main task can positively enhance subsequent sports related performance by 1.31%–12% (Ng et al., 2020; Finlay et al., 2022b). Regarding combat sports, the efficacy of this mode of CA was shown to enhance specific performances in judo (Miarka et al., 2011; Lum, 2019), karate (Margaritopoulos et al., 2015), and taekwondo (Da Silva Santos et al., 2015; Ouergui et al., 2022). In fact, Margaritopoulos et al. (2015) reported that performing three sets of five tuck jumps followed by 5 min of rest improved the jumping performance of karate athletes by 3.5%. Moreover, Da Silva Santos et al. (2015) showed that taekwondo athletes improved their number of kicks in a specific task when using a CA based on complex exercise (i.e., combined strength and plyometric exercise: half-squat 3 × 2 at 95% 1 R M + 4 vertical jumps over a 40-cm barrier) when a 10-min rest interval was used compared to the control condition. In addition, Ouergui et al. (2022) found that three sets of 5-s repeated jumps above 40 cm followed by 10 min of rest, enhanced the kicking performances of taekwondo athletes when the effort-pause ratio (E:P) within the CA was 1:7 and self-selected. In grappling combat sports; Miarka et al. (2011) reported that 10 sets of three consecutive jumps over increased obstacles (i.e., 20, 40 and 60 cm) performed 3 min before the Special Judo Fitness Test improved the number of throws by 12% in male judo athletes. Moreover, Lum (2019) reported that CA based on explosive exercises for upper and lower body (ULB) (i.e., warm-up followed two sets of five repetitions of resistance band pull at maximal effort and two sets of five repetitions of standing broad jump) or for lower body (LB) (i.e., warm-up followed by three sets of five repetitions of standing broad jump) performed 7 min before the Special Judo Fitness Test improved the number of throws during series 1 compared to control condition, while only ULB increased the total number of throws in male judo athletes. The use of combat sport-specific actions is also reported to be an efficient activity prescribed during training programs (Franchini, 2020). Indeed, specific techniques have been used to prepare athletes to effectively manage the physical and physiological demands of their sport (Ouergui et al., 2020), and acutely augment power production (Finlay et al., 2022a). In the PAPE context, the use of technical-based exercises has been reported to stimulate the neuromuscular system (Cimadoro et al., 2019) and enhance performance in striking contests (Aandahl et al., 2018; Ouergui et al., 2022). Interestingly, Aandahl et al. (2018) showed that three sets of 10 roundhouse kicks resisted by elastic induced significant improvement in kick speed by 3.3% and muscular activity by 35.2%–43.9% in mixed group of combat sports athletes following a rest interval of five to 8 min. However, this type of activity has not received much interest as a conditioning activity to induce PAPE in combat sports (Ouergui et al., 2022), which implies the need of further study.

Empirical research indeed supports the efficacy of explosive-type CA exercises in inducing PAPE (Boullosa, 2021), albeit this is not a consistent observation within the literature (Da Silva Santos et al., 2016; Castro-Garrido et al., 2020). In fact, Da Silva Santos et al. (2016) did not report beneficial effect on taekwondo specific performance 10 min of rest after performing a plyometric based CA, which is the same result reported by Castro Garrido et al. (2020) even when different modes of CA were used (i.e., isometric, plyometric and contrast). The inconsistent findings are generally associated, but not limited, to different factors including CA mode, rest intervals between CA and the subsequent test, volume, intensity, and subject characteristics (Tillin and Bishop, 2009; Wilson et al., 2013). Although activity type and loading variables are the CA moderators that have received most attention (Boullosa, 2021), many areas require further exploration, especially, considering the duration of stimulus and rest between sets (i.e., effort to pause ratio, E:P) and its interaction with other moderators. Previous studies have examined the influence of E:P in relation to the magnitude of the potentiation (Ouergui et al., 2022), but using only a single rest interval between the conditioning activity and subsequent tests (i.e., 10 min). There remains considerable debate surrounding the rest time needed to optimize potentiation effects following CA, and a lack of scientific evidence concerning its interaction with the CA modes that can serve to enhance performance optimally (Dobbs et al., 2019). Indeed, 7 min is typically considered as the common rest interval suggested to elicit a PAPE effect (Wilson et al., 2013; Dobbs et al., 2019). In terms of the latter, CA that is energetically and biomechanically similar to the technical actions performed in the sport might modulate the level of potentiation (Hodson-Tole and Wakeling, 2009).

PAPE has been widely suggested as an efficient strategy to improve athletic performance (Blazevich and Babault, 2019). However, this phenomenon is very sensitive to several factors including rest interval, CA mode, characteristics of populations, and the E:P ratio within the CA (Seitz and Haff, 2016; Ouergui et al., 2022). To the best of our knowledge, no empirical study has investigated the effect of manipulating the E:P ratio, the mode of CA, and the rest interval between CA and the main task, as well as their interactions on subsequent performance. Therefore, this study was designed to assess these deficiencies, by examining the influence of two modes of CA (i.e., plyometric and repeated high-intensity techniques), performed using different E:P ratios (i.e., 1:6, 1:9, self-selected rest, SSR), and rest intervals (i.e., 3 and 7 min) on taekwondo jump height, specific agility and the frequency and speed of kick performances. It was hypothesized that: 1) incorporating repeated high-intensity techniques (RT) and plyometric P) conditioning activities into a taekwondo warm-up would result in better performance when compared with a control condition; 2) CA effects will be modulated by the E:P and rest interval, and there will be interaction effects.

## 2 Materials and methods

### 2.1 Participants

An *a priori* power analysis was calculated using the G*Power software (Version 3.1.9.4, University of Kiel, Kiel, Germany) using the F test family (ANOVA: repeated measures, within factors) to determine the required sample size with α set at 0.05 and power (1-β) set at 0.80. The analysis revealed that a total sample size of 13 subjects would be sufficient to find significant differences (effect size f = 0.25, α = 0.05) with an actual power of 83.62%. To guarantee statistical power and prevent the unfortunate occurrence of participant dropouts, twenty-one Tunisian taekwondo athletes (males = 13 and females = 8) volunteered to participate in the present study. The descriptive characteristics of participants are presented in Table 1. Following a convenience sampling, they were recruited from the same training club based on the following inclusion criteria: age >18 years, participating in the same training regime (3 times per week, 2 h per session), having at least 5 years of training experience. Athletes present any medical restrictions during the experimental period including any lower body injury or any neuromuscular disorder were excluded from the study. Athletes were asked to follow the same diet, avoid strenuous exercises, and restrain from any ergogenic aid or stimulant 48 h prior to each session. This study was conducted according to the Declaration of Helsinki for human experimentation and approved by a local research ethics committee (CPP SUD N◦ 0332/2021). A written informed consent was obtained after a detailed explanation about the aims and risks involved in the investigation.

**TABLE 1:** Descriptive characteristics of the participants (Values are mean ± SD; n = 21).

Age (years)	Height (cm)	Body mass (kg)	Experience (years)
20.4 ± 1.4	170.9 ± 9.3	61.6 ± 9.7	6 ± 1

### 2.2 Procedures

**FIGURE 1:**
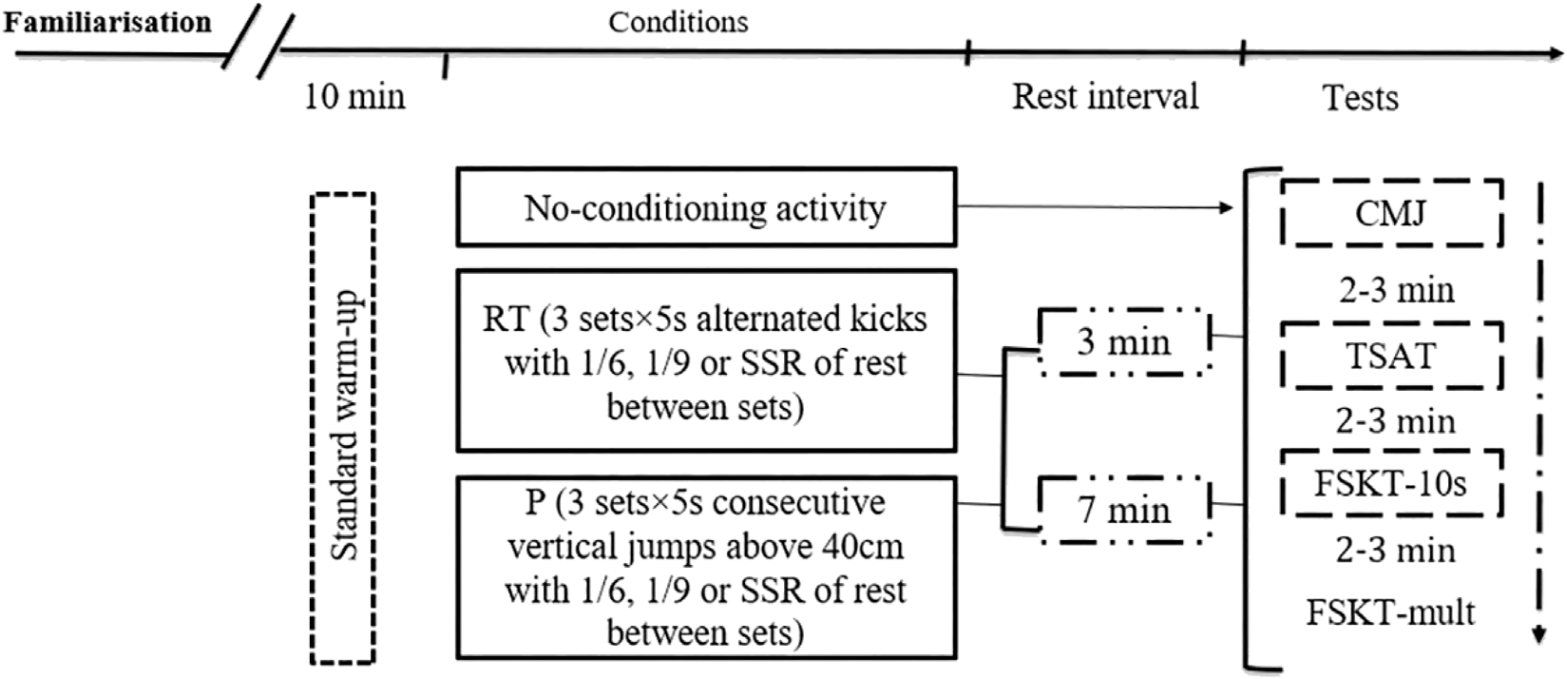
Schematic representation of the study design. RT: Repeated techniques; P: Plyometric; SSR: Self Selected Rest; CMJ: Counter-movement Jump; TSAT: Taekwondo Specific Agility Test; FSKT-10s: 10s Frequency speed of kick test; FSKT-mult: multiple frequency speed of kick test.

The present study describes some procedures that were previously published by (Ouergui et al., 2022). Although both studies are part of a larger research project, some differences should be highlighted. Precisely, the present study recruited other participants with different characteristics compared to the previous one. Additionally, despite using a similar CA, various intrinsic components were manipulated. These included the rest periods between the CA and subsequent tests (i.e., 10 min in (Ouergui et al., 2022) *versus* 3 and 7 min in the present study) and the inclusion of a new, larger E:P ratio (i.e., 1:7 in the previous study *versus* 1:9 in the present one) within the CA. This is a repeated-measures study design where athletes participated in 12 experimental conditions resulting from the combination between rest interval (3 min or 7 min), effort to pause ratios (1:6, 1:9 and self-selected rest) and activity type (repeated high-intensity techniques and Plyometric) and a control condition (Figure 1). One week before the beginning of the experimentation, the characteristics of the participants were recorded and athletes were familiarized with the testing procedures (i.e., tests and different conditions). Specifically, in the first session, athletes were interviewed regarding their age, years of practice, and weekly hours of training. Additionally, the athletes were weighed using a bioimpedance analyzer (BF679W; Tanita, Japan), and their height measured usinga stadiometer (Seca model 213, Germany) in a bipedal position. After that, athletes performed a control condition (i.e., consisting of 10 min standard warm-up followed by 2 min of rest (Da Silva Santos et al., 2015) and 11 experimental conditions in a randomized cross-over order. Concerning experimental conditions, after accomplishing the same standard warm-up as performed during habitual training sessions (i.e., 5 min of running at an average speed of 9 km/h + 5 min taekwondo specific activities), athletes performed, as much as they could, repeated-high intensity techniques [i.e., three sets of alternate kicks, using the bandal-chagui technique (i.e., a technique of striking the target by swinging the foot inward while the supporting leg does not pivot), executing the maximum of kicks possible over 5 s]or plyometric (i.e., three sets of consecutive vertical jumps using both legs as fast as possible over an obstacle of 40 cm for 5 s) using different E:P ratios within each CA (i.e., 1:6, 1:9 and SSR), with three or 7 min of rest interval between the conditioning activity and the subsequent tests. The choice of 3 and 7 min as rest intervals was based on previous meta-analyses results (Wilson et al., 2013; Dobbs et al., 2019) that showed performance enhancement at rest intervals of 3–7 min range. The testing sessions were performed in separate days with a 48-h interval between consecutive sessions. After the control and experimental conditions, athletes performed, in the same order, the countermovement jump test (Markovic et al., 2004) to assess jump height, the taekwondo specific agility test (TSAT) (Chaabene et al., 2018) for specific agility, the 10 s frequency speed of kick test (FSKT-10s) and its multiple form (FSKT-mult) (Da Silva Santos et al., 2015; Da Silva Santos et al., 2016) to evaluate the number of techniques executed, with a rest period of 2–3 min between the consecutive tests. The tests were performed at 17–18 h for all sessions to avoid any diurnal variation of the performance.

#### 2.2.1 Countermovement jump test

From a standing position, athletes performed a fast downward movement by flexing the knees and hips immediately followed by a rapid extension of these joints while keeping their hands on their waists. No lower limb flexion or arm swing in the upward phase was allowed. The CMJ test was performed using an infrared jump system (Optojump, Microgate, Bolzano, Italy). Three trials were carried out, and the best performance was maintained for analysis. The intra-class correlation coefficient (ICC) for test–retest trial for the present study was 0.98.

#### 2.2.2 Taekwondo specific agility test

From his/her fighting stance position and behind the start line, the athlete freely advanced to the center mark as fast as possible. Then, at will, the athlete turned towards partner one using a shift motion and performed a roundhouse kick with their lead leg, turned to the other side, shifted to partner 2, and performed another roundhouse kick with the other lead leg. Thereafter, he/she returned to the center, moved to partner three in guard position, and performed a double roundhouse kick. Finally, the athlete ran backward to the start/finish line (Chaabene et al., 2018). The sparring partners maintained the kick-target at the trunk height of the tested athlete. The performance time was measured with two sets of single-beam timing lights (Brower Timing Systems, Salt Lake City, UT, United States). Each athlete performed three trials, and the best performance was used for the analysis. The ICC for test-retest trial for the present study was 0.93.

#### 2.2.3 Ten seconds frequency speed of kick test

During the FSKT-10s test, along the 10 s bout each athlete performed the maximum number of kicks (i.e., bandal-chagui) against a bag atthetrunkheightby alternating the right and left lower limbs, with the total number of kicks representing the performance in this test (Da Silva Santos et al., 2015). The ICC for test-retest trial for the present study was 0.82.

#### 2.2.4 Multiple frequency speed of kick test

Each athlete performed five sets of FSKT-10s with a 10 s rest interval between repetitions (Da Silva Santos et al., 2016). Performance was determined as the total number of kicks performed in each set and the total number of kicks in five sets, which was used for the subsequent analysis. The ICC for test-retest trial for the present study was 0.77.

#### 2.2.5 Statistical analysis

Data were presented as mean and SD. The statistical analysis was performed using SPSS 20.0 statistical software (IBM corps, Armonk, NY, United States). The normality of data sets was checked and confirmed using the Shapiro-Wilk test. Sphericity was tested and confirmed using the Mauchly test and a Greenhouse-Geisser correction was used when necessary. To compare the control condition with different experimental conditions, data were analyzed using a one-way analysis of variance with repeated measurements to compare performances. Moreover, a three-way analysis of variance [Rest (3 and 7 min) × effort-to-pause ratio (1:6, 1:9 and SSR) × activity type (RT and P)] with repeated measurements was used to compare experimental conditions. When an interaction effect was found, this was the only result reported for a given variable. Bonferroni was used as post-hoc test and partial eta squared (η_p_^2^) was used as effect size. Moreover, standardized effect size (Cohen’s d) analysis was used to interpret the magnitude of differences between variables and classified according to Hopkins (2002): d ≤ 0.2 (trivial), 0.2 < d ≤ 0.6 (small), 0.6 < d ≤ 1.2 (moderate), 1.2 < d ≤ 2.0 (large), 2.0 < d ≤ 4.0 (very large), and d > 4.0 (extremely large). The statistical significance level was set at *p* ≤ 0.05.

## 3 Results

### 3.1 CMJ performance between conditions

**FIGURE 2:**
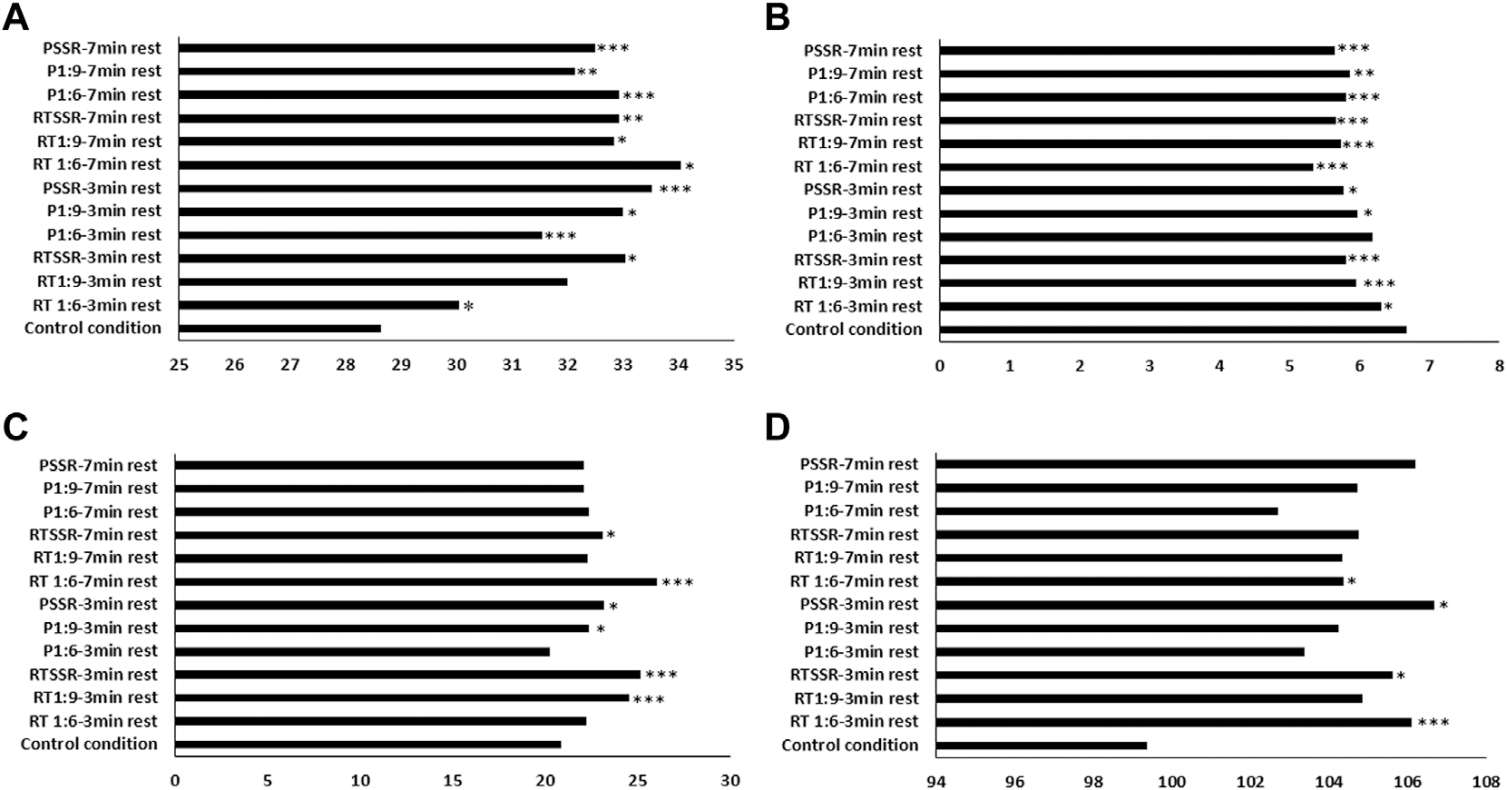
**(A)** Countermovement jump (cm) **(B)** agility (seconds) **(C)** 10 s (number) and **(D)** multiple frequency speed kick tests performances (number) recorded during experimental conditions compared to control condition. * different from control condition at *p* < 0.05, **: different from control condition at *p* < 0.01, *** different from control condition at *p* < 0.001. P: plyometric; RT: repeated high-intensity techniques; SSR: self-selected rest.

There was a significant difference between conditions (F_3.45,69.10_ = 101.405; *p* < 0.001; η_p_^2^ = 0.342), with the control condition eliciting lower values than RT condition using 1:6 and SSR ratios with 3 min of rest (95%CI = -2.3;-0.4 and -7.4;-1.3; both *p* = 0.001; d = -0.19; and -0.67, respectively), P condition using 1:6, 1:9 and SSR ratios using 3 min of rest (95%CI = -4.8;-1.0; -7.8;-0.9 and -7.7;-1.9; *p* < 0.001; *p* = 0.005 and *p* < 0.001; d = -0.43; -0.71 and -0.76, respectively), RT condition using 1:6, 1:9 and SSR ratio using 7 min of rest (95%CI = -9.4;-1.3; -8.2;-0.2 and -7.1;-1.4; *p* = 0.003; *p* = 0.034 and *p* = 0.001; d = -0.90; -0.70 and -0.65, respectively) and P condition using 1:6, 1:9 and SSR ratio using 7 min of rest (95%CI = -6.7;-1.9; -7.8;-1.1 and -5.9;-1.8; *p* < 0.001; *p* = 0.001 and *p* < 0.001; d = -0.66; -0.53 and -0.57, respectively) (Figure 2).

### 3.2 TSAT performance between conditions

There was a significant difference between conditions (F_5.32,106.31_ = 16.373; *p* < 0.001; η_p_^2^ = 0.450), with the control condition resulting in lower performance that RT condition with 1:6, 1:9 and SSR ratio using 3 min of rest (95%CI = 0.03; 0.7; 0.4; 1.1 and 0.4; 1.3; *p* = 0.024, both *p* < 0.001; d = 0.62; 1.35 and 1.74, respectively), P with 1:9 and SSR ratios using 3 min of rest (95%CI = 0.1; 1.2 and 0.2; 1.6; *p* = 0.005 and *p* = 0.004; d = 1.48 and 1.87, respectively), RT condition with 1:6, 1:9 and SSR ratio using 7 min of rest (95%CI = 0.8; 1.8; 0.4; 1.4 and 0.4; 1.6; all *p* < 0.001; d = 2.63; 1.82 and 1.92, respectively) and P condition with 1:6, 1:9 and SSR ratio using 7 min of rest (95%CI = 0.3; 1.4; 0.2; 1.3 and 0.4; 1.6; *p* < 0.001; *p* = 0.001 and *p* < 0.001; d = 1.68; 1.47 and 2.03, respectively) (Figure 2).

### 3.3 FSKT10s and FSKT-mult performance between conditions

For FSKT10s, there was a significant difference between conditions (F_12,240_ = 36.076; *p* < 0.001; η_p_^2^ = 0.661), with control condition inducing lower values compared to RT condition using 1:9 and SSR ratios with 3 min of rest (95%CI = -5.1;-2.2 and -6.0;-2.6; both *p* < 0.001; d = -2.56 and -3.33, respectively), P condition using 1:9 and SSR ratio with 3 min of rest (95%CI = -2.9;-0.9 and -4.3;-0.4; *p* = 0.029 and *p* = 0.008; d = -1.07 and -1.43, respectively), RT condition using 1:6 and SSR ration with 7 min of rest (95%CI = -6.9;-3.5 and -3.8;-0.7; *p* < 0.001 and *p* = 0.001; d = -3.64 and -1.63, respectively) (Figure 2).

Regarding FSKT-mult, there was a significant difference between conditions (F_5.24,104.80_ = 3.385; *p* = 0.006; η_p_^2^ = 0.145), with control condition eliciting lower techniques’ number than RT condition using 1:6 and SSR ratio with 3 min of rest (95%CI = -10.8;-2.6 and -12.0;-0.5; *p* < 0.001 and *p* = 0.023; d = -1.18 and -1.15, respectively), P condition using SSR ratio with 3 min of rest (95%CI = -14.0;-0.5; *p* = 0.024; d = -1.14) and RT condition using 1:6 ratio with 7 min of rest (95%CI = -9.7;-0.3; *p* = 0.030; d = -0.89) (Figure 2).

Table 2 presents the results of the jump height and agility performances during different experimental conditions.

**TABLE 2:** Countermovement jump (CMJ) and agility (TSAT) performances recorded during experimental conditions (Values are mean ± SD; *n* = 21).

CA	Rest	Ratio	CMJ (cm)	TSAT (s)
RT	3 min^a^	1:6	30.1 ± 7.4^b,c,f,g,j^	6.31 ± 0.56
		1:9	32.0 ± 5.2^k^	5.94 ± 0.50
		SSR	33.1 ± 5.6	5.80 ± 0.40^†^
P	3 min^a^	1:6	31.6 ± 6.1^b^	6.20 ± 0.30
		1:9	33.0 ± 4.5^h^	6.0 ± 0.30
		SSR	33.5 ± 5.3	5.80 ± 0.32^†^
RT	7 min	1:6	34.0 ± 4.1^i,l^	5.34 ± 0.40
		1:9	32.9 ± 4.2	5.74 ± 0.40
		SSR	32.9 ± 5.5	5.70 ± 0.43^†^
P	7 min	1:6	32.9 ± 5.4	5.80 ± 0.42
		1:9	32.1 ± 5.7^days^	5.90 ± 0.50
		SSR	32.5 ± 6.0^e^	5.65 ± 0.40^†^

^a^ main effect of rest interval: 3 min induced lower CMJ values than 7 min (*p* = 0.011); b interaction effect between rest and ratio: 1:6 ratio with 3 min rest induced lower values than 7 min rest and than 1:9 and SSR ratio using 3 min of rest (*p* = 0.001); c interaction effect between rest, ratio and activity type: RT condition using 1:6 ratio induced lower values using 3 min compared to 7 min of rest (*p* = 0.011), d interaction effect between rest, ratio and activity type: P condition using 1:9 ratio induced lower values with 7 min compared to 3 min of rest (*p* = 0.007), e interaction effect between rest, ratio and activity type: P condition using SSR ratio induced lower values with 7 min compared to 3 min of rest (*p* = 0.032), f interaction effect between rest, ratio and activity type: RT with 3 min of rest induced lower values with 1:6 compared to 1:9 ratio (*p* = 0.002), g interaction effect between rest, ratio and activity type: RT with 3 min of rest induced lower values with 1:6 compared to SSR ratio (*p* = 0.010), h interaction effect between rest, ratio and activity type: P using 3 min of rest induced lower values with 1:9 compared to SSR ratio (*p* = 0.009), i interaction effect between rest, ratio and activity type: RT using 7 min of rest induced higher values with 1:6 compared to SSR ratio (*p* = 0.043), j interaction effect between rest, ratio and activity type: 1:6 with 3 min of rest induced lower values with RT compared to P condition (*p* = 0.037), k interaction effect between rest, ratio and activity type: 1: 9 ratio using 3 min of rest induced higher values with RT compared to P condition (*p* = 0.027), l interaction effect between rest, ratio and activity type:1: 6 ratio using 7 min of rest induced higher values with RT condition compared to P (*p* = 0.016); † main effect of ratio: SSR ratio induced better performance than 1:6 (*p* = 0.038) and 1:9 ratios (*p* = 0.021); RT: repeated high-intensity techniques; P: plyometrics; SSR: self-selected rest; CA: conditioning activity.

### 3.4 CMJ performance main effects and interactions between CA type, rest and ratios

For the CMJ test, there was a significant interaction effect between rest and ratio (F_1.35,27.10_ = 10.193; *p* = 0.002; η_p_^2^ = 0.338), with the ratio 1:6 using 3 min of rest inducing lower values than 7 min of rest (95%CI = -3.7;-1.1; *p* = 0.001; d = -0.46) and ratio 1:6 eliciting lower values than 1:9 and SSR ratio using 3 min of rest (95%CI = -2.1;-0.5 and -3.6;-0.9; both *p* = 0.001; d = -0.29 and -0.41, respectively). Moreover, there was a significant interaction effect between rest, ratio and activity type (F_2,40_ = 7.695; *p* = 0.001; η_p_^2^ = 0.278), with RT condition using 1:6 ratio resulting in lower values using 3 min compared to 7 min of rest (95% CI = -6.0;-1.9; *p* = 0.011; d = -0.67), and P condition using 1:9 ratio inducing lower values with 7 min compared to 3 min of rest (95%CI = -2.3;-0.4; *p* = 0.007; d = -0.17) and P condition using SSR ratio elicited lower values with 7 min compared to 3 min of rest (95%CI = -1.9;-0.1; *p* = 0.032; d = -0.18). Moreover, RT condition with 3 min of rest induced lower values with 1:6 compared to 1:9 and SSR ratios (95%CI = -4.9;-1.1 and -5.3;-0.6; *p* = 0.002 and 0.010; d = -0.30 and -0.46, respectively), and P condition using 3 min of rest resulted in lower values with 1:9 compared to SSR ratio (95%CI = -3.5;-0.4; *p* = 0.009; d = -0.1), and RT condition using 7 min of rest induced higher values with 1:6 compared to SSR ratio (95%CI = 0.05; 3.8; *p* = 0.043; d = 0.23). Furthermore, the ratio 1:6 with 3 min of rest elicited lower values with RT compared to P condition (95%CI = -3.8;-0.1; *p* = 0.037; d = -0.22) and 1:9 ratio using 3 min of rest elicited higher values with RT compared to P condition (95%CI = 0.2; 2.8; *p* = 0.027; d = 0.21). Additionally, 1:6 ratio using 7 min of rest resulted in higher values with RT condition compared to P (95%CI = 0.2; 2.1; *p* = 0.016; d = 0.23).

### 3.5 TSAT performance main effects and interactions between CA type, rest and ratios

For the TSAT performance, there was a significant main effect of ratio (F_2,40_ = 4.265; *p* = 0.021; η_p_^2^ = 0.176), with SSR ratio inducing better performance (i.e., lower time) compared to 1:6 and 1:9 ratios (95%CI = -0.4;-0.01 and -0.3;-0.02; *p* = 0.038 and *p* = 0.021; d = -0.37 and -0.40, respectively).

Table 3 presents the total number of kicks recorded during FSKT-10s and FSKT-mult during different experimental conditions.

**TABLE 3:** Ten seconds and multiple frequency speed kick tests performances recorded during experimental conditions (Values are mean ± SD; *n* = 21).

CA	Rest (min)	Ratio	FSKT-10s (n)	FSKT-mult (n)
RT	3	1:6	22 ± 1^a,c,e,g,i,j,k,l^	106 ± 5
		1:9	25 ± 1^b,c,h,i,j,k,l^	105 ± 5
		SSR	25 ± 1^c,e,j,k,†,^ˠ	106 ± 4
P	3	1:6	20 ± 2^a,e,g,k,l,φ,¶^	103 ± 6
		1:9	22 ± 1^b,h,k,†,¥^	104 ± 7
		SSR	23 ± 2^e,k,^ˠ	107 ± 7
RT	7	1:6	26 ± 1^a,c,e,g,i,£^	104 ± 5
		1:9	22 ± 1^b,c,d,f,h,i,j^	104 ± 5
		SSR	23 ± 1^c,e,j,£,l^	105 ± 5
P	7	1:6	22 ± 1^a,e,g,£,¶^	103 ± 7
		1:9	22 ± 1^b,d,f,h,l^	105 ± 8
		SSR	22 ± 2^e,£^	106 ± 8

^a^ Main effect of ratio: 1:6 different from SSR ratio (*p* = 0.005); b main effect of ratio: 1:9 different from SSR ratio (*p* < 0.001); c main effect of activity type: RT different from plyometrics (*p* < 0.001). d interaction effect between ratio and rest: 1:9 ratio induced higher values than 1:6 and SSR ratios using 7 min of rest (*p* < 0.001); e interaction effect between ratio and rest: 1:6 and SSR ratios after 3 min different from after 7 min (*p* < 0.05); f interaction effect between ratio and rest: 7 min induced better results compared to 3 min using 1:9 ratio (*p* < 0.001); g interaction effect between ratio and activity type: different from RT with SSR (*p* = 0.001); h interaction effect between ratio and activity type: different from RT with SSR (*p* < 0.001); i interaction effect between ratio and activity type: different from P in 1:6 and 1:9 ratios (*p* < 0.001); j interaction effect between rest and activity type: different from P with 3 and 7 min (*p* < 0.001); k interaction effect between rest and activity type: different from 7 min for both RT and P (*p* < 0.001); i interaction effect between rest, ratio and activity type: different from 7 min for RT condition with 1:6 and 1:9 and P with 1:6 (*p* < 0.001); † interaction effect between rest, ratio and activity type: different from 7 min for P with 1:9 and RT with SSR ratios (*p* < 0.05); ˠ interaction effect between rest, ratio and activity type: different from RT using 3 min of rest with 1:9 ratio (*p* = 0.034); φ interaction effect between rest, ratio and activity type: different from P activity using 3 min of rest with SSR (*p* = 0.028); ¥ interaction effect between rest, ratio and activity type: different from P activity using 3 min of rest with SSR (*p* < 0.001); £ interaction effect between rest, ratio and activity type: different from RT and P activities using 7 min of rest with 1:9 ratio (*p* < 0.001); interaction effect between rest, ratio and activity type: different from RT condition with 3 min and 7 min of rest using 1:6 ratio (*p* < 0.001); RT: repeated high‐intensity techniques; P: plyometrics; SSR: self‐selected rest; CA: conditioning activity; FSKT‐10: ten seconds frequency speed of kick test; FSKT‐mult: multiple frequency speed of kick test.

### 3.6 FSKT-10 s and FSKT-mult performance main effects and interactions between CA type, rest and ratios

For FSKT-10, there was a significant interaction effect between ratio and rest (F_2,40_ = 18.958; *p* < 0.001; η_p_^2^ = 0.487), with 1:9 ratio inducing higher values than 1:6 and SSR ratios using 7 min of rest (95%CI = 0.9; 2.0 and 1.5; 2.6; both *p* < 0.001; d = 1.12 and 0.32, respectively) and better performances after 3 min compared to 7 min of rest using 1:6 and SSR ratios (95%CI = both 0.1; 1.2; *p* = 0.025 and *p* = 0.032; d = -1.50 and -1.02, respectively), while 7 min elicited better results compared to 3 min of rest using 1:9 ratio (95%CI = 0.9; 1.9; *p* < 0.001; d = -0.88, respectively). Likewise, there was a significant interaction effect between ratio and activity type (F_1.50,30.01_ = 5.598; *p* = 0.014; η_p_^2^ = 0.219), with 1:6 and 1:9 inducing better performance than SSR ratio using RT condition (95%CI = 0.4; 1.8 and 0.9; 2.2; *p* = 0.001 and *p* < 0.001; d = -2.79 and -0.67, respectively) and RT condition resulted in better results than P in 1:6 and 1:9 ratios (95%CI = 0.9; 1.7 and 1.0; 2.0; both *p* < 0.001; d = -1.41 and -1.84, respectively). Likewise, there was a significant interaction effect between rest and activity type (F_1,20_ = 154.093; *p* < 0.001; η_p_^2^ = 0.885), with RT resulting in lower values than P condition using 3 min of rest (95%CI = -1.1;-0.4; *p* < 0.001; d = -1.15) and RT induced higher values than P condition using 7 min of rest (95%CI = 2.4; 3.4; *p* < 0.001; d = 0.98). Moreover, 3 min elicited lower values than 7 min of rest with RT condition (95%CI = -2.4;-3.4; *p* < 0.001; d = -0.08), while 3 min induced higher values than 7 min of rest with P condition (95%CI = 1.4; 2.4; *p* < 0.001; d = 0.13). There was a significant interaction effect between rest, ratio and activity type (F_2,40_ = 69.649; *p* < 0.001; η_p_^2^ = 0.777), with RT condition using 1:6 and 1:9 ratios inducing lower values with 3 min of rest compared to 7 min (95%CI = -3.6;-2.3 and -4.4;-3.0; both *p* < 0.001; d = -3.15 and -1.96, respectively), P condition using 1:6 and 1:9 ratios eliciting higher values with 3 min compared to 7 min of rest (95%CI = 3.4; 5.1 and 0.2; 1.6; *p* < 0.001 and *p* = 0.018; d = 1.55 and 0.22, respectively) and RT condition using SSR ratio resulting in higher values with 3 min compared to 7 min of rest (95%CI = 0.2; 1.8; *p* = 0.014; d = 2.12). Moreover, the RT condition using 3 min of rest induced lower values with 1:9 compared to SSR ratio (95%CI = -1.5;-0.02; *p* = 0.043; d = -0.67) and that P activity using 3 min of rest resulted in higher values with SSR compared to 1:6 and 1:9 ratios (95%CI = 0.1; 2.5 and 1.3; 3.0; *p* = 0.028 and *p* < 0.001; d = 1.88 and 0.58, respectively). Additionally, RT activity using 7 min of rest induced higher values with 1:9 compared to 1:6 and SSR ratios (95%CI = 0.1; 1.6 and 3.1; 4.8; both *p* < 0.001; d = 3.33 and 0.77, respectively) and RT activity using 7 min of rest elicited higher values with 1:6 compared to SSR ratio (95%CI = 2.2; 3.9; *p* < 0.001; d = 2.56). Furthermore, P activity using 7 min of rest induced lower values with 1:6 compared to 1:9 and SSR ratios (95%CI = -2.9;-1.1 and -2.8;-0.8; both *p* < 0.001; d = -0.19 and -0.17, respectively). Likewise, values after 3 min of rest using 1:6 were higher with P compared to RT activity (95%CI = 1.6; 2.9; *p* < 0.001; d = 1.42) while RT condition induced higher values with 7 min of rest using 1:6 ratio compared to P (95%CI = 1.6; 2.9; *p* < 0.001; d = 3.28). Moreover, 3 min of rest using 1:9 ratio induced higher values with P compared to RT condition (95%CI = 0.01; 1.5; *p* = 0.034; d = 1.84), while RT condition induced higher values with 1:9 ratio using 7 min of rest compared to P condition (95%CI = 3.0; 4.5; *p* < 0.001; d = 0.15). Finally, 3 min of rest using SSR ratio induced higher values with RT compared to P condition (95%CI = 0.0; 1.5; *p* = 0.034; d = 1.58).

For FSKT-mult, there was an interaction effect between ratio and activity type (F_2,40_ = 3.826; *p* = 0.030; η_p_^2^ = 0.161), however the Bonferroni post-hoc did not detect any difference (*p* > 0.05).

## 4 Discussion

The present study investigated the effects of manipulating the activity type (RT and P), rest interval (3 or 7 min), and the E:P ratio (1:6, 1:9 and SSR) on the specific physical performance of taekwondo athletes. The main findings of the present study confirmed partially our hypotheses. In fact, performances in all the tests were better following CAs as compared to the control condition. Moreover, during CMJ and FSKT-10s, there were clear interactions between rest interval and E:*p*, influencing the efficacy of the CA. In fact, P favors 3 min rest regardless of the E:P whereas RT favors 7 min, especially, when 1:6 and 1:9 ratios were used.

In the present study, the effectiveness of including CA within the warm-up confirmed previous findings, which reported that using such intervention induces PAPE in taekwondo specific performances (Da Silva Santos et al., 2015; Ouergui et al., 2022). However, since PAPE is a volatile phenomenon and is not necessarily linked to improved performance (MacIntosh et al., 2012), these results were not unanimous (Da Silva Santos et al., 2016; Castro-Garrido et al., 2020). These inconsistent findings could be linked to inter-individual variability and the CA′ modulating factors (e.g., mode, rest, E:P ratio, volume) (Tillin and Bishop, 2009; Wilson et al., 2013). This could be evident in the case of CMJ, as using similar CA mode with different rest intervals or E:P ratios did not improve jump height in previous studies (Da Silva Santos et al., 2015; Ouergui et al., 2022). Whilst the CMJ is considered to lack mechanical specificity to the technical actions performed in the sport, and it is a very short task to reflect PAPE benefit (Da Silva Santos et al., 2015; Castro-Garrido et al., 2020; Ouergui et al., 2022), the CA’ metrics (i.e., rest intervals, within effort-to-pause ratios and activity types) in our study demonstrated its potential in augmenting power production in the lower limbs. Since PAPE could be compliant with the theory of neural enhancement (Blazevich and Babault, 2019), the jump height was improved following CA warm-up (i.e., RT and P) because, most likely, the neural system enhanced the upward movement of the center of mass in the vertical jump by activating multi-joint muscles (Voigt et al., 1995). Regarding the improved kicking performance recorded in the present study, our results were similar to a previous study using kicks with elastic resistance as a CA followed by 5–8 min rest period (Aandahl et al., 2018). In fact, this enhanced kicking performance reflected greater kicking velocity mediated in part through a greater transmission of power to the moving structure, better synchronization of the involved motor units, and reduced pre-synaptic inhibition (Aandahl et al., 2018).

The current study demonstrated that the CA’s efficacy was modulated by its E:P ratio and the rest interval before the subsequent task. In fact, greater performances were observed during the CMJ and FSKT-10s after 3 min of rest when a self-selected rest ratio was used within both modes of CA (i.e., RT and P). Therefore, this result could suggest that during a CA, it is more adequate to give the opportunity to athletes to choose their own rest time between sets which will result in better performances when short rest intervals are planed prior to the main test. The fact that SSR was better than specific ratios (i.e., 1:6 and 1:9) could be associated with high inter-individual differences in PAPE/fatigue relationship (do Carmo et al., 2021). The key benefit of SSR over fixed rest may be that it enables athletes to choose when they are most confident to perform the subsequent task, which increases the chance of PAPE occurring (do Carmo et al., 2021). However, in previous study, Da Silva Santos et al. (2015) reported that taekwondo athletes were not able to self-determine the best moment to induce PAPE. The evidence provided from this later study was concerning the rest interval between CA and the main task, but not between CA sets, which might indicate that the E:P ratio has similar, if not greater, moderating effect as the rest interval has.

It is widely recognized that the time interval between CA and the main exercise has a direct influence on the relationship between potentiation and fatigue (Borba et al., 2017). In particular, it has been documented that performance may be improved if potentiation dominates fatigue, maintained if potentiation and fatigue are at equal levels, or decreased if fatigue dominates potentiation (Seitz and Haff, 2016). In the current study, it was found that repeated high-intensity techniques resulted in greater effect during CMJ and FSKT-10s at 7 min with the 1:6 and 1:9 ratios than it did with SSR, whereas plyometrics induced greater performance at 3 min compared to 7 min regardless of the ratios. Previous meta-analyses (Wilson et al., 2013; Dobbs et al., 2019) have reported that rest intervals ranging from about 3 to 7 min had more potentiating effects than intervals longer than 10 min. This was explained by the fact that when periods were relatively brief, fatigue would overwhelm the potentiation process, lowering performance, and when periods were very prolonged, potentiation would disappear (MacIntosh et al., 2012). The association between PAPE and fatigue was dependent on the rest intervals and the E:P ratios, which allowed the current investigation to provide insight into the relativeness of these fluctuations. The fact that P-based CA followed by 3 min resulted in greater performances compared to after 7 min was similar to previous findings (Seitz and Haff, 2016). In fact, it has been shown that P CA may cause low exhaustion, enabling a strong potentiation effect while shortening the time required (0.3–4 min) for the optimal PAPE benefit (Seitz and Haff, 2016). This is consistent with previous research using elite karate athletes where a 5-min rest interval resulted in performance enhancement following plyometric intervention (Margaritopoulos et al., 2015). Plyometric exercises are useful tool for boosting tendon stiffness and enhancing the lower body’s jump and strength capabilities (Ramirez-delaCruz et al., 2022). Reasonably, plyometric exercises are associated with the recruitment of preferentially type II motor units, which, in turn, enhance neural stimulation when high power outputs are required (Chen et al., 2013). The findings of the current study highlight that the explosive-type loading used in this experiment improved the recruitment of fast-twitch units and neuromuscular system function, without causing excessive fatigue (Masamoto et al., 2003). Regarding the use of specific technique, a larger rest interval and more specific ratios were required to induce PAPE. Notably, RT is a specific competitive explosive technique activity that may solicit greater activation of musculature (Cimadoro et al., 2019), and induce greater fatigue, compared to what resulted after the P activity. Using 1:9 and 1:6 ratios, RT was better than P at 7 min of rest, while at 3 min of rest, P was better than RT. These findings could lead athletes to use preferentially fixed ratios over SSR when the selected rest interval was appropriate to the mode of conditioning activity. In this regard, it is critical to note that some participants may not be able to find the “sweet spot” during SSR time if they relax for an excessive period and thereby down-regulate PAPE (Wilson et al., 2013).

Although PAPE has been reported to be optimal at an interval of 3–10 min (Wilson et al., 2013; Dobbs et al., 2019), the present study showed that its effect persisted over consecutive tasks (i.e., >10 min at least for FSKT-mult). Such result could indicate that short-duration contractions would not allow fatigue to build up during contractions, while still promoting PAPE (Rassier and Macintosh, 2000). This could be evident since the given rest interval between tests would allow muscle recovery, enhancing the prevalence of PAPE over fatigue (Batista et al., 2007). PAPE may occur as a result of the alteration in the stimulation pattern of motor units, resulting in an increase in the number of excited motor units (Borba et al., 2017). This effect of activation history on the subsequent force production might be greater when subsequent exercise simulate the previously activated motor units (Hodson-Tole and Wakeling, 2009; Arabatzi et al., 2014). Since the tests used in the present study commonly stimulated the lower limbs muscle groups, the promotion of PAPE could indicate a PAPE accumulation over the tasks when an adequate rest interval was used (Batista et al., 2007).

Existing evidence indicates that the specificity of the potentiating activity and how much it corresponds to the subsequent exercise may influence PAPE in different athletic activities (Tillin and Bishop, 2009). To this end, conditioning activity replicating the mechanical actions and muscle contractions experienced during the event should conceivably evoke more efficient/specialized recruitment of motor units (Hodson-Tole and Wakeling, 2009). Based on these reports, it could be postulated that plyometrics would more effectively potentiate motor unit recruitment patterns specific to the task of vertical jump and plyometrics, and thereby elicit suboptimal stimulus to the muscle being tasked with the FSKT-10s performance. Nevertheless, improved performance was recorded in the FSKT-10s in the current investigation even though P was not task dependent to kicking performance. This improvement can be explained by the fact that round kick force is significantly and positively correlated with lower limb performance metrics (i.e., CMJ height, power, relative power and force) (Loturco et al., 2014). The training status of the participants should also be taken into consideration when interpreting the results (Margaritopoulos et al., 2015; Boullosa, 2021). Given the connection between potentiation, fatigue, and strength, resistance trained individuals could better withstand fatigue after the CA and reach their maximum PAPE response earlier than less well-trained individuals (Seitz and Haff, 2016). In the meta-analysis conducted by Dobbs et al. (2019), the sensitivity analysis showed that excluding untrained individuals resulted in shorter optimal rest interval in trained ones. In the present study, the participants were well-trained athletes, and therefore needed a shorter rest interval to achieve their optimal performance in the task after the conditioning activity (Tillin and Bishop, 2009). While the CAs resulted in better performances compared with control, the fact that no difference between CAs was found for TSAT and FSKT-mult could suggest that neuromuscular fatigue was similar (Cimadoro et al., 2019). Probably, during tasks like the FSKT-mult, performance enhancement required longer rest interval (e.g., 10 min) as reported in previous investigation (Ouergui et al., 2022). However, Castro-Garrido et al. (2020) did not report improvements when the same rest period was used (i.e., 10min), which may be explained by the fact that the P CA was used without manipulating E:P ratio, suggesting that PAPE is a delicate stimulus which requires an interconnection between the different factors that modulate CA’s effects. In addition, the inter-individual variables such as training status, muscle fiber type, training age, sex, and strength level, as mentioned earlier, can have an impact on PAPE (Robbins, 2005; Tillin and Bishop, 2009; Wilson et al., 2013; Seitz and Haff, 2016). This inter-individual variability may especially influence how individuals respond to a stimulus, with some showing positive responses while others may not (Robbins, 2005). It is worth noting that the data are typically analyzed on a group level rather than individually, which may explain the limited detection of PAPE in certain studieswith small and heterogeneous sample sizes (e.g., Castro-Garrido et al. (2020).

Agility, kicking speed and power are among the physical qualities required to cope successfully with taekwondo combat demands (Bridge et al., 2014). Therefore, their improvement can implicate an increase in combat intensity. Moreover, the techniques used during the FSKT-10s and its multiple version are among the most used techniques during an official competition (Kazemi et al., 2006). Consequently, it is possible to suggest that agility and kicking performance enhancement could result in more technical exchanges and scoring actions during the 2-min round of taekwondo combat. Although the present study could have important implications for athletes, coaches, and scientists seeking to improve taekwondo short-term performance, there were some limitations that should be acknowledged. In fact, the physiological mechanisms that are proposed to take place as a result of PAPE were not measured. Moreover, although significant differences were found between the control and experimental conditions for all the tests, the use of consecutive tests may have caused fatigue and limited the PAPE effect. Finally, despite using taekwondo-specific tests, they may not fully reflect the stress induced by an official match. Therefore, considering these limitations, it is recommended to incorporate CA in more realistic match-like scenarios and explore additional factors that may influence the effects of CA (e.g., fitness level, duration of CA, and the main task). Furthermore, future studies are encouraged to investigate the neurophysiological mechanisms underlying PAPE.

## 5 Conclusion

This study showed that incorporating plyometric and repeated high-intensity techniques activities into taekwondo warm-up induced positive effects on the kicking performance, specific agility and jumping height for taekwondo athletes. The integration of a CA using a SSR time within sets resulted in performance enhancement with short rest interval, and this could be used during taekwondo training sessions targeting power development. Moreover, using P activity required shorter rest interval as compared to repeated high-intensity techniques to induce PAPE. Such effects could deem it beneficial to include plyometric as part of a pre-competition warm-up. Additionally, using specific ratios within CA resulted in better performances following both CAs. Thus, mimicking the load of taekwondo combat by using specific E:P ratios could determine the level of potentiation. Likewise, the PAPE effect with repeated high-intensity techniques-based CA required longer time to appear than with P. Therefore, increasing the number of training sessions in which athletes receive a PAPE stimulus may enhance the long-term adaptations as well as the likelihood of a positive transfer of training sessions to competitive settings. Generally, P and RT CAs require minimal logistics, and activate several muscles groups simultaneously (Cimadoro et al., 2019; Ng et al., 2020). Therefore, they could be more convenient methods to induce PAPE and improve athletic performances during training and competition.

## Data Availability

The raw data supporting the conclusion of this article will be made available by the authors, without undue reservation.
